# Biological sources of inflexibility in brain and behavior with aging and neurodegenerative diseases

**DOI:** 10.3389/fnsys.2012.00077

**Published:** 2012-11-30

**Authors:** S. Lee Hong, George V. Rebec

**Affiliations:** ^1^Department of Biomedical Sciences, Ohio UniversityAthens, OH, USA; ^2^Department of Psychological and Brain Sciences, Indiana UniversityBloomington, IN, USA

**Keywords:** aging, neurodegenerative diseases, neural noise, dopamine, glutamates

## Abstract

Almost unequivocally, aging and neurodegeneration lead to deficits in neural information processing. These declines are marked by increased neural noise that is associated with increased variability or inconsistency in behavioral patterns. While it is often viewed that these problems arise from dysregulation of dopamine (DA), a monoamine modulator, glutamate (GLU), an excitatory amino acid that interacts with DA, also plays a role in determining the level of neural noise. We review literature demonstrating that neural noise is highest at both high and low levels of DA and GLU, allowing their interaction to form a many-to-one solution map for neural noise modulation. With aging and neurodegeneration, the range over which DA and GLU can be modulated is decreased leading to inflexibility in brain activity and behavior. As the capacity to modulate neural noise is restricted, the ability to shift noise from one brain region to another is reduced, leading to greater uniformity in signal-to-noise ratios across the entire brain. A negative consequence at the level of behavior is inflexibility that reduces the ability to: (1) switch from one behavior to another; and (2) stabilize a behavioral pattern against external perturbations. In this paper, we develop a theoretical framework where inflexibility across brain and behavior, rather than inconsistency and variability is the more important problem in aging and neurodegeneration. This theoretical framework of inflexibility in aging and neurodegeneration leads to the hypotheses that: (1) dysfunction in either or both of the DA and GLU systems restricts the ability to modulate neural noise; and (2) levels of neural noise and variability in brain activation will be dedifferentiated and more evenly distributed across the brain; and (3) changes in neural noise and behavioral variability in response to different task demands and changes in the environment will be reduced.

## Introduction

Generally, as people age, their movement and cognitive patterns become more variable. Widely termed “behavioral inconsistency,” age-related declines in motor and cognitive function are indexed as an increased magnitude of variability; for example, a higher standard deviation in response times around an average (Williams et al., 2005). A leading explanation is that with age comes an increase in neural noise, that is, a greater occurrence of random neural activity (Li et al., 2001; Li and Sikström, 2002). Recent findings, however, have begun to challenge the idea of a direct positive correlation between increased variability in brain activity and increased behavioral variability. Another aspect of neural noise that continues to evolve is the dysfunction of key neurotransmitters that may give rise to noisy brain activity and its ensuing effects on behavior. Dopamine (DA), which modulates neuronal excitability in striatum and other forebrain areas that control motor and cognitive functions [for a review see Graybiel (2000)] has always been a focal point of the discussion in aging, neurodegeneration, and neural noise.

Relevant to this point, Li et al. (2001) used mathematical modeling to suggest that the increased neural noise in aging arises from a decline in DA function. While the loss of DA neurons is widely viewed as the biological mechanism underlying increased neural noise in aging (Li et al., 2001; Li and Sikström, 2002; Macdonald et al., 2012), multiple complications are evident. For one, the transmission of neural information is negatively affected by excessively high as well as low DA activity (Stewart and Plenz, 2006), making it difficult to view DA loss as the exclusive underlying mechanism. From a therapeutic standpoint, moreover, the most widely used strategy for increasing DA function is administration of L-DOPA, the immediate DA precursor, which is only marginally effective and plagued by motor and other untoward side effects (e.g., Carey et al., 1995; Merims and Giladi, 2008; Gerlach et al., 2011). But perhaps more importantly, DA is unlikely to play the sole or even lead role in neural noise. Growing evidence, for example, suggests that neural noise can arise from decreased (or perhaps blocked) uptake of glutamate (GLU) (Arnth-Jensen et al., 2002; Tzingounis and Wadiche, 2007), an excitatory amino acid transmitter. This finding is particularly relevant to the study of geriatrics and gerontology because GLU uptake declines with aging (Segovia et al., 2001).

Furthermore, there is a growing body of literature that proposes that aging is a central component in the development of many neurodegenerative disorders, including Parkinson's (PD) (Collier et al., 2011), Alzheimer's (AD) (Duncan, 2011; Barron and Pike, 2012), and Huntington's (HD) (Vonsattel et al., 2011; Jiang et al., 2012) diseases as well as Essential Tremor (Louis, 2009). Effectively, neurodegenerative disorders are viewed to be reflections of pathological deviations from the trajectory of normal aging to a point at which age-related declines are compounded, leading to the manifestation of clinical symptoms that increase in severity over time. As a result, it is quite likely that at least some of the neurobiological mechanisms of decline in neurodegenerative disorders are shared with those of normal aging.

The goal of this theory-and-hypothesis paper is to develop a framework by synthesizing the literature surrounding the role of DA and GLU in neural noise and behavioral variability in both aging and neurodegeneration. We will highlight areas of convergence in the aging and neurodegeneration literature in order to provide specific neurobiological explanations for changes in neurobehavioral variability. Most often, increased variability in cognitive and motor performance is termed “behavioral inconsistency.” In this paper, we will present a view that behavioral “*inflexibility*,” rather than inconsistency, that is, the inability to: (A) resist external perturbations and maintain performance on a single task; and (B) adapt to different task demands; is the central problem of aging and neurodegeneration. We raise the possibility that DA and GLU have a shared role in modulating neural noise, allowing for a many-to-one solution map to control noise levels in the brain. We then discuss the behavioral implications of an impaired ability to distribute levels of neural noise and variability in brain activity. Finally, we propose that aging and neurodegeneration lead to inflexibility at three levels of analysis: (1) neurotransmitter function; (2) brain activation; and (3) behavior. The ensuing result is a failure to adapt to changing task demands and environmental conditions.

## Neural noise and DA neuromodulation in aging

An oft-cited mechanism for increased behavioral inconsistency with aging is the neural noise hypothesis, in which aging leads to increased random activation patterns in the brain (e.g., Allen et al., 1992; Nesselroade and Salthouse, 2004). Although noise in the nervous system arises at a variety of different levels from a variety of different sources (Faisal et al., 2008), it has been hypothesized that a decline in DA function underlies the biological basis for age-related increases in neural noise (Li et al., 2001).

Neural noise is also a common factor in a variety of neurodegenerative disorders beyond that of normal aging such as AD, HD, and PD. Increased neural noise is evident in HD, appearing as a decrease in burst activity and a loss of correlation between the firing patterns of pairs of neurons in the striatum of 6–9 week-old R6/2 transgenic HD mice (Miller et al., 2008b). In this age range, R6/2 mice are in the early stages of symptom development, prior to the widespread neuron loss caused by HD (Cepeda et al., 2007). In fact, R6/2 mice show relatively little neuron loss even as symptoms worsen. These and other lines of evidence raise questions as to whether neurodegeneration and cell loss are a central component of the behavioral symptoms or if dysfunctional activation patterns can give rise to HD symptoms (André et al., 2010). Thus, a potential common factor in aging and neurodegeneration is that neural noise and dysfunctional neural communication is central to clinical and subclinical declines at the level of behavior. In fact, a breakdown in neural communication and increased neural noise might be an early sign of an impending broader loss of neurons.

A widely documented phenomenon of the aging process is a decline in the number of DA neurons: 10% are lost with each decade of life after the age of 20 years (Wong et al., 1997). The loss of DA alters neuronal sensitivity to GLU, changing the “gain” in the input-activation relationship such that neurons in the elderly generate a lower output per unit of input (Li et al., 2001; Li and Sikström, 2002). A decline in DA, therefore, diminishes the strength of the GLU signal above background activity (Rebec, 2000). In forebrain neurons, which receive both DA and GLU input, a diminished signal-to-noise ratio can impair both motor and cognitive functions (Kiyatkin and Rebec, 1996).

Support for an age-related decline in DA transmission comes from analysis of post-mortem tissue [see Reeves et al. (2002) for a review] and from *in vivo* methods, such as single photon emission computed tomography (SPECT) and positron emission tomography (PET) in humans or microdialysis in animals. At best, however, these approaches provide an incomplete picture in that they can only measure the baseline or steady state level of extracellular DA (Reeves et al., 2002), which results from the slow, pacemaker firing of DA neurons (Grace, 2000). On top of this tonic level of DA activity, DA neurons generate phasic activity of transient clusters or bursts of spikes in close association with a wide range of behaviorally relevant events, including the presentation of salient or rewarding stimuli (e.g., Fiorillo et al., 2003; Schultz, 2010). Thus, behavior is shaped by a complex interaction of both tonic and phasic DA activity. Relating behavioral events to phasic DA signals, however, is difficult and requires the use of electrophysiological techniques to assess DA firing patterns or fast-scan cyclic voltammetry to detect DA release events on a sub-second time-scale while behavioral responses are carefully monitored (Garris and Rebec, 2002). Unfortunately, these experimental approaches have not been widely used in the study of aging or neurodegenerative disease.

Additional complications include the inherent compensatory mechanisms that help to maintain a healthy level of DA transmission despite an aging- or disease-related loss of DA neurons (Collier et al., 2011). Compensation for a decline in DA release, for example, may include an increase in sensitivity of post-synaptic DA receptors or a slower rate of clearance of DA from the synapse. Although these mechanisms may keep DA transmission within a normal range, they are unable to keep up with circumstances that place increasing demands on the system. Thus, as the number of ways in which DA signaling can respond to events decreases, the DA response becomes increasingly inflexible. Under these conditions, DA begins to lose its effectiveness in modulating neural noise.

A decrease in DA is not the only way in which neural transmission becomes dysfunctional. When DA is excessively high, natural correlated firing patterns begin to break down (Stewart and Plenz, 2006). In fact, there is an optimal level of DA transmission that is most conducive for the transfer of neural information when tested *in vitro* (Surmeier et al., 2011). When DA is too low, the spatial correlations in electrical activity across neural networks break down, but a similar phenomenon occurs when DA is excessively high such that the electrical activity in the network becomes uncorrelated. This inverted-U shaped relationship between DA transmission and network activity shows that neural activity patterns become increasingly unpredictable and random when synaptic DA is either too high or too low [also see Williams and Castner (2006)].

## Role of dopamine in behavioral changes due to aging and neurodegeneration

Increased variability in cognitive response time as a function of age is thought to result from increased neural noise that leads to behavioral inconsistency (Allen et al., 1992; Li et al., 2001, 2006). Indeed, numerous studies report increased response times in seniors when compared to young adults (e.g., Williams et al., 2005). Similar age-related increases in variability are also observed in motor behavior such that old adults generally perform with increased error and greater variability. Studies of postural control (e.g., Sheldon, 1963) and gait patterns (e.g., Kurz and Stergiou, 2003) show that larger magnitudes of sway and variability in stride length and time are indicative of an aging-related decline in motor control. In short, there is converging evidence that increased variability is associated with declining neural function.

Similar to its effects on brain patterns, excessively high and low DA can lead to undesired patterns of behavior, as reviewed in Hills (2006). When synaptic DA levels are too high, behavioral patterns become stereotypical and perseverative. On the other hand, synaptic DA levels that are too low lead to patterns of behavior that are unfocused or scattered. In aging, it is generally believed that declines in DA transmission result in declines in cognitive and motor performance (Li et al., 2001; Li and Sikström, 2002; Düzel et al., 2010) that manifest as increased behavioral inconsistency. In HD, however, the role of DA in shaping the behavioral phenotype is not as clear. Most often, choreatic movements are viewed to be the result of increased DA transmission and are treated with DA-blocking agents, while akinesia is thought to result from a loss of DA function [see André et al. (2010) for a review]. This view is consistent with clinical evidence that tetrabenazine, which depletes brain DA, can improve the adventitious movements that appear early in the course of HD (Hayden et al., 2009). In contrast, the most consistent finding in rodent models is a decrease in phasic DA release (Johnson et al., 2006), but it will be important to assess DA release in these models early in symptom development when there is evidence of hyperkinesia.

In human HD patients, walking on a treadmill results in more random and uncorrelated patterns of gait variability in comparison to controls (Hausdorff et al., 1997). From one step to the next, control subjects show more consistent and sequential changes in variability in stepping patterns than the HD patients. A similar disruption of stepping patterns during gait is also evident in transgenic mice that model HD (Fowler et al., 2009). Interestingly, elderly subjects also show a similar decline in stride-to-stride correlations. In fact, aging (Hausdorff et al., 1997) and PD (Hausdorff et al., 1998) also lead to a similar breakdown in correlations between walking strides. This uncorrelated or scattered pattern of gait variability seems to be prevalent in both aging and neurodegeneration.

It is interesting to note that deficits in gait patterns in HD subjects examined by Hausdorff et al. (1997) were not associated with specific motor symptoms. Instead, increasing randomness in gait had a stronger relationship with poorer Total Functional Capacity (TFC) scores on the Unified Huntington's Disease Rating Scale (UHDRS). What this finding suggests is that the increased behavioral randomness is evidence of more generalized underlying neural dysfunction, as opposed to being a specific deficit to the neuromotor system. While we cannot ascertain *post-hoc* the degree to which the DA system was impaired in the HD subjects tested in Hausdorff et al. (1997), it is likely that relative to young controls, the behavioral data converge with those of the aging and PD subjects, which would implicate excessively low DA levels. Based on prevailing viewpoints (Li et al., 2001; Hills, 2006), the evidence is suggestive of low DA levels leading to increased neural noise, which results in the scattered and unfocused gait patterns in HD, PD, and aging.

## Glutamate and neural noise

Although not often the center of discussion on the topic of neural noise in aging, a high level of synaptic GLU also increases neural noise. Arnth-Jensen et al. (2002) provided a key example by assessing the effects of GLU uptake blockade on activity in hippocampal CA1 pyramidal cells. When GLU uptake was impaired by DL-threo-β-benzyloxyaspartate (TBOA), which blocks multiple varieties of GLU transporters (Shimamoto et al., 1998). Arnth-Jensen et al. (2002) observed an increase in the noisy fluctuations in the excitatory post-synaptic currents of CA1 cells stimulated by an external source. There is evidence that aging leads to a decrease in GLU uptake, particularly in cerebral cortex and striatum (Wheeler and Ondo, 1986). Some studies using rat models observed a 20–30% age-related decline in GLU uptake and a 70–80% in mice, although the evidence remains equivocal to a large extent, due in part to differences in methodology and measurement [see Segovia et al. (2001) for a review].

Beyond increased neural noise, when GLU uptake is restricted, high synaptic levels of GLU increase neural excitability (Tzingounis and Wadiche, 2007), which in turn would flatten the probability distributions of neural activity, not unlike the effects of a decline in DA transmission presented in Li et al. (2001). Due to the decrease in GLU uptake in aging, if both young and old individuals have a similar level of GLU release, then a decrease in GLU uptake would mean an increase in extracellular GLU in the elderly [see McEntee and Crook (1993) for a review]. High extracellular GLU can trigger neurodegeneration by activating extra-synaptic NMDA receptors (Okamoto et al., 2009; Milnerwood et al., 2010). In fact, excess GLU has been implicated as a potential risk factor in AD (Hynd et al., 2004), PD (Levy et al., 2009), and HD (Estrada-Sánchez and Rebec, 2012).

One result of increased extracellular GLU could be a compensatory decline in the operation of GLU receptors. In fact, a consistent finding in research on aging animals is a decrease in the density (Wenk et al., 1991; Magnusson and Cotman, 1992; Nicolle et al., 1996) of N-methy-D-aspartate (NMDA) receptors, which mediate key aspects of GLU-mediated plasticity (Muller et al., 1994). These receptors also lose responsiveness to GLU. For example, NMDA receptor-mediated effects on synaptic responses, membrane excitability, and generation of action potentials decline as mammals age (Cepeda et al., 1996). Changes in NMDA receptor function are likely to play a major role in the cognitive declines that occur with aging. Alpha-amino-3-hyrdoxy-5-methyl-4-isoxazole-propionic acid (AMPA) and kainate receptors, which are the workhorses of GLU-mediated fast excitatory transmission, also show evidence of down-regulation with aging, but this effect is not as robust as the NMDA receptor change (Segovia et al., 2001).

Two complications make it difficult to arrive at a definitive conclusion regarding aging-related effects on GLU transmission. The first is the extent of the compensatory changes that occur in any neuronal system when one component is altered. A change in receptors in response to a decline in uptake is just one example. In addition, release could also change. In fact, a decrease in uptake could be a compensatory response to a decline in release. Teasing the various components apart is difficult because of the second complication: limitations in measuring extracellular GLU. Conventional microdialysis, which is the method on which most assessments of GLU transmission are based, depends on analyzing a sample of extracellular fluid collected over the course of several minutes (Timmerman and Westerink, 1997). This means that the level of extracellular GLU is really a balance of both release and uptake. Most importantly, however, the bulk of extracellular GLU may not even be a direct result of neuronal activity, coming instead from a cysteine-GLU exchange mechanism related to the control of oxidative stress (Baker et al., 2002). Thus, GLU transmission is likely changing in multiple ways with aging. Although a loss of GLU uptake accounts for one of these changes, the critical issue is how the change in GLU transmission interacts with aging-related changes in the DA system to increase neural noise.

Altered GLU transmission poses a problem in the aging process due to deficits in compensatory increases in the release of other neurotransmitters, DA in particular. As reviewed in Segovia et al. (2001), blocking GLU uptake in the striatum and nucleus accumbens of young rats leads to the release of GABA and DA. In elderly rats, the DA response to increased GLU is blunted in the nucleus accumbens. Interestingly, the DA-GLU relationship in aged rats in striatum remains unchanged. Thus, if a young and old rat were to have similar synaptic levels of endogenous GLU, neurons in the aged rat would be unable to release an appropriate amount of DA. This becomes a particular problem as neural noise levels due to high levels of extracellular GLU cannot be attenuated by a concomitant increase in DA. It could be argued that this effect of aging is one example of decreased flexibility in the ability to modulate neural noise levels through the DA-GLU relationship.

## Glutamate in behavior

GLU also has a role to play in the behavioral deficits that emerge in aging and neurodegeneration. For example, elevated synaptic GLU triggered by a decrease in GLU uptake or a failure of one or more additional mechanisms are proposed to be important factors in AD and have a role to play in deficits in learning and memory (McEntee and Crook, 1993). As a potential treatment for PD, antagonists of post-synaptic GLU receptors have been suggested as a possible means of improving motor function (Starr et al., 1997). A less dramatic means of decreasing the synaptic efficacy of GLU in PD could include an increase in GLU uptake (Chung et al., 2008). This mechanism appears to work in HD. Treatment with β-lactam antibiotic ceftriaxone improves both GLU uptake and behavioral signs in transgenic R6/2 mice (Miller et al., 2008a). The behavioral improvement is evident as an increase in motor flexibility, seen in a greater number of 90° turns made in a plus maze. In fact, ceftriaxone treatment resulted in increased expression of GLT, the protein responsible for the bulk of GLU uptake in striatum.

As with DA, excessively high or low levels of GLU pose problems in that neural noise changes with the synaptic level of GLU also forms a U-shaped function. When GLU receptor function is blocked by memantine, a treatment for AD, motor stereotypies result (Kos and Popik, 2005). This effect is similar to that of excessively high levels of DA, which leads to perseveration and repetitive or stereotypical patterns of behavior (Hills, 2006). Consistent with the DA-GLU relationship proposed by Segovia et al. (2001), it is possible that DA and GLU both play a role in modulating neural noise levels in the brain.

If DA and GLU are theorized as lying within a limited scope and restricted to a single pathway and mechanism of action, they can only serve as neurotransmitters and sources of noise. Their combined interaction on the other hand, engenders the DA-GLU system with the capacity to serve the function of noise modulation. But, based on their molecular characteristics, DA and GLU act along different pathways and mechanisms leading to different kinetics. Furthermore, although DA neurons project from the midbrain to as far as frontal cortex and the cerebellar peduncle [see Björklund and Dunnett (2007) for a recent review], they are not omnipresent in the brain. The question that arises is how these seemingly disparate systems with an incongruity in structure and function could play the role of neuromodulator through their interactions.

In a complex system with many components such as the brain, direct, individualized control of every structure is virtually impossible. A mathematical model developed by Gelfand and Tsetlin (1971) provides the foundation for the manner in which individual structures can form functional units through a principle of minimal interaction or minimal afferentation. Effectively, direct input to each individual component is not necessary, insofar that these individual components interact to form a cohesive network (Gutman, 1994). There is further support for the Gelfand–Tsetlin model in the forces produced by the fingers of the human hand. The strategy of coordinated control of the finger forces are not governed by anatomy or the biomechanics of the hand alone (Latash et al., 1998).

**Figure 1:**
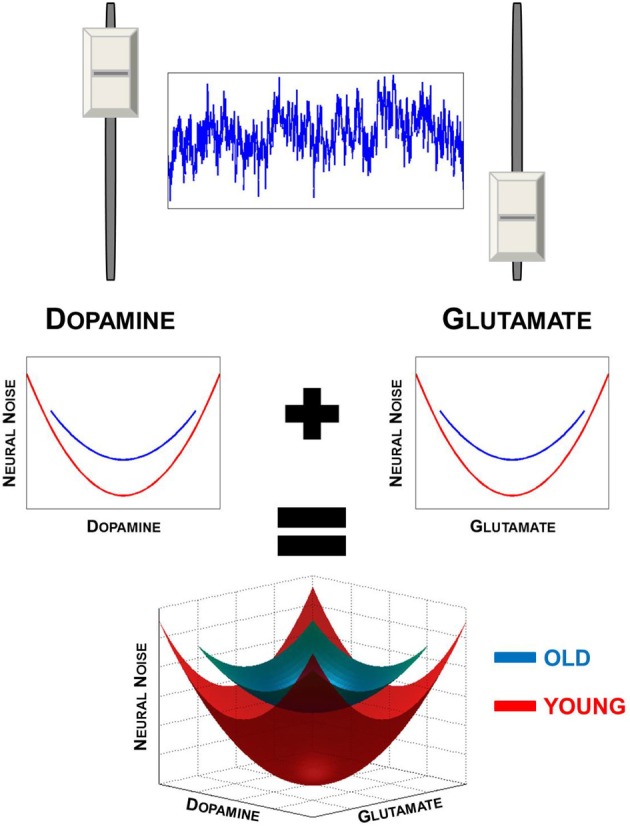
**Degeneracy in neural noise modulation.** Upper panel illustrates that both GLU and DA have an effect on neural noise levels. Noise is highest when both DA and GLU levels are either too high or too low. Lower panel provides an illustration of the U-shaped the relationship DA and GLU have with neural noise (upper panels). When these two functions are combined, a 3-dimensional solution surface or manifold is formed (lower panel), illustrating the degeneracy in neural noise modulation. Also illustrated is the effect of aging, which results in the narrowing of the range of DA and GLU levels in the elderly. This results in a smaller solution manifold, restricting the range of neural noise levels in the old.

As a result, the central nervous system can be both stable and flexible (Gelfand and Tsetlin, 1971; Gutman, 1994) by functioning under simple rules and organizing the many components into functional units that are not constrained by structural and anatomical limitations. What this allows is for “many-to-one” mapping between structure (i.e., DA and GLU neurons and receptors) and function (i.e., neural noise modulation) supports the concept of degeneracy in neuroanatomy and cognitive function (Edelman and Gally, 2001; Price and Friston, 2002; Noppeney et al., 2004). It is particularly advantageous to involve two neurotransmitters in setting neural noise levels. DA and GLU work on different time-scales allowing a broader dynamic range over which noise can be modulated (Figure 1, upper panel).

Apart from the flexibility in which noise levels can be modulated, another benefit of degeneracy within the DA-GLU systems is that similar levels of neural noise can be achieved using different combinations of DA and GLU (Figure 1, lower panel). One can envision this as a solution manifold or surface, along which different levels of DA and GLU combine to generate a given level of neural noise based on the summation of two U-shaped functions. The independence of DA and GLU from an anatomical and structural standpoint is advantageous as both neurotransmitters act along different pathways. This affords greater flexibility by allowing noise to be modulated by DA and GLU on a broader range of spatial and temporal scales. Additionally, the different kinetics and dynamics of DA and GLU would mean that they would not have to act within a direct negative feedback (agonist-antagonist) loop to one another. For lack of a better analogy, this system is very much like having a car with two independent brake-accelerator systems (where DA and GLU can each act as accelerator and brake), one running and electric motor and one controlling the gas motor. Each would have different magnitudes and rates of action in controlling the speed of the vehicle, which in the brain would be analogous to neural noise levels. For example, as DA is being taken up and noise is increasing on a slow time-scale (i.e., accelerator-off), GLU is able to act to quickly either to speed up or halt the increase in noise (i.e., speed of the car). What this allows is for rapid phasic changes to be made when needed, even against the backdrop of slower tonic modulation of neural noise, leading to the complex dynamics observed in the brain. With aging, one would expect that deleterious effects of aging and neurodegeneration arise due to a decrease in DA release (Li et al., 2001; Li and Sikström, 2002; Düzel et al., 2010), a decline in GLU uptake (Segovia et al., 2001; Chung et al., 2008), altered GLU transmission (Morrison and Baxter, 2012), and an altered relationship between DA and GLU (Segovia et al., 2001; André et al., 2010).

One of the most consistent findings in the aging mammalian brain is a decline in D2-like DA receptor binding, especially in striatum (Hoekzema et al., 2011), which receives DA input from the substantia nigra, the pathway that degenerates in PD (Collier et al., 2011). The loss of D2-like receptor activity in old age may be as much as 50% of the young adult (Antonini et al., 1993). Although less information is available on age-related changes in the D1-like receptor, it appears that D1 receptor loss is fairly widespread, occurring in striatal, limbic, and cortical areas (Rieckmann et al., 2011). Thus, cognitive tasks that depend on working memory and tasks related to goal-directed behavior are likely to be affected by dysregulation of DA transmission. Interestingly, the density of dendritic spines on cortical neurons, which receive both GLU and DA innervation, also declines with aging (Morrison and Baxter, 2012). The strength of synaptic input onto spines, moreover, is modulated by cyclicAMP-protein kinase A signaling, which regulates K+ channel conductance to control neuronal firing patterns (Chen et al., 2001; Wang et al., 2011). Impairments in this signaling mechanism are a likely explanation for age-related loss of cognitive and motor function (Morrison and Baxter, 2012).

Taken as a whole, these negative effects of aging and neurodegeneration on DA and GLU anatomical structures and transmission result in a system that is inflexible as there would be fewer available solutions to set neural noise levels in the brain. Furthermore, the range over which neural noise can be modulated in time and space would be also restricted. This can be captured as a shrinking solution manifold in which a smaller range of synaptic DA and GLU restricts the range of neural noise that can be achieved (Figure 1, lower panel).

## Should neural noise always be associated with greater variability in brain activation?

Variability has generally been viewed as a marker of decreased function based on the concept of homeostasis, in which maintaining a physiological function or output around a mean would indicate stability. Thus, a variable physiological output is viewed as a negative, indicating the presence of undesired fluctuations around a steady state. While a sizeable proportion of the literature points to neural and behavioral variability as markers of poor brain health, there is a growing body of literature that suggests the contrary, i.e., that variability serves a functional, adaptive purpose (Lipsitz and Goldberger, 1992; Lipsitz, 2002; Neuringer, 2002; Vaillancourt and Newell, 2002). One of the difficult aspects of studying physiological signals is separating them from noise (Glass, 2001). Since the underlying signal is often unknown, unpredictability, and noise often have to be made synonymous as the underlying signal is difficult to separate empirically from random background activity (Pincus, 1991; Beltrami, 1999; Glass, 2001).

Thus, one of the options for testing the role of neural noise is to use models of the nervous system for which the underlying signal is pre-determined and can be directly separated from noise. Li et al. (2006) used a model to test whether ideal levels of background noise could benefit the transmission of neural signals based on the concept of stochastic resonance. Effectively, stochastic resonance refers to a situation in which the addition of a small amount of noise or random variation leads to a change in the behavior of a non-linear system (Gammaitoni et al., 1998; Moss et al., 2004). Li et al. (2006) used an extension of the mathematical model the same research group presented in 2001, but with added external noise to test the effects of aging. This model showed that “young” neurons with higher DA input and thus, higher gain, were more efficient, requiring a lower level of background noise to produce the desired output. These results show that a greater amount of external noise has to be added to the already randomly firing “old” neurons in order for the same signal to be transmitted appropriately.

## Do more variable brain activation patterns necessarily lead to behavioral inconsistency?

The results of the model used in Li et al. (2006) suggest that variability or noise in brain activation does not necessarily lead to poorer signal transmission. However, in order to move from model to empirical data, ensuing studies have raised the question as to whether internal brain noise could play a functional role in cognitive performance measured at the behavioral level. If neural noise indeed serves a functional purpose, the elderly would actually benefit from greater underlying variability in their brain activation patterns (Garrett et al., 2010). Recent findings from neuroimaging research demonstrate that seniors who perform more slowly and inconsistently exhibit more variable brain activation over the course of a series of repeated cognitive tasks, taken at 2-s intervals (Garrett et al., 2011). This finding would suggest that variability in brain activation and inconsistent behavioral responses are not necessarily synonymous.

Greater variability in brain activity alone, as indexed by the BOLD signal, however, was not the only distinguishing characteristic for cognitive performance. Rather, high performing individuals exhibited a high degree of variability in the brain regions that accounted for a majority of the total variance across the brain, but exhibited very low levels of variability in the remaining (primarily subcortical) regions. Interestingly, the brains of seniors that performed more poorly were not only less variable overall, but also exhibited dedifferentiated levels of variability across both regions that accounted for a high and low proportion of the total BOLD signal variance. Once again, this finding raises the issue that greater variability in brain activity alone is not always: (A) an indicator of increased neural noise; and (B) a marker of ill health.

## Is greater behavioral variability a necessary indicator of aging and neurodegeneration?

One important issue that has been raised is whether patterns of behavioral variability should immediately be dismissed as noise (Newell et al., 2006), just like variable brain activity in the BOLD signal obtained from an fMRI (Garrett et al., 2010). It is also argued that the increased noise levels within the brain during maturation are reflections of the expansion of the “dynamic repertoire” that increases the functionality of neural networks (McIntosh et al., 2010). In old adults, McIntosh et al. (2010) show a reconfiguration of regional noise, but, the general relationship between brain noise and stable performance is maintained, consistent with Garrett et al. (2011). Effectively, these results suggest that the ability to shift noisy brain activity from one region to another is important for a high level of cognitive function. Furthermore, healthy brain function is not indicated by a universally low level of variability in brain activity. Instead, brain health is reflected by high levels of variability in certain regions and low levels of variability in others.

Much like noise in the brain, one of the core concepts of human motor behavior is the idea of signal-dependent noise in movement execution. Put simply, larger neural signals or motor commands are required to generate larger muscle forces for either more powerful or faster movements. With noise that is signal-dependent, a proportional increase in noise is expected, which in turn leads to motor variance (Fitts and Peterson, 1964; Schmidt et al., 1979; Harris and Wolpert, 1998). Since neural noise is expected to increase in aging, it is has always been a logical hypothesis to predict observable increases in motor variability in the elderly.

Indeed, as mentioned earlier, increased variability in aging has been observed across a range of motor tasks. Similar to the problem of variability in brain function, increased motor variability in aging and neurodegeneration cannot in and of itself be linked to neural noise and randomness. Two individuals with the same magnitude of variability could exhibit drastically different patterns of fluctuations when unfolded over time. What distinguishes healthy and unhealthy fluctuations in heartbeat intervals is the fact that healthy individuals exhibit more irregular heartbeat patterns that include both fast and slow fluctuations (Lipsitz and Goldberger, 1992). On the other hand, the unhealthy pattern is characterized by a more regular pattern of fluctuation restricted to fewer and slower time-scales of change. This type of evidence resulted in the creation of the “loss of complexity” framework that placed greater emphasis on the dynamics or time-dependent sequences as well as the shape of the data distribution, rather than magnitude alone (Lipsitz, 2002; Vaillancourt and Newell, 2002; Newell et al., 2006). In sum, patterns of physiological output that are only correlated on very short time-scales and random patterns that are uncorrelated over time are indicators of ill health from the complexity theory approach.

One of the common problems with the study of patterns of variability in behavior is that these patterns are fundamentally dependent on task demands and environmental conditions [see Newell (1986); Vaillancourt and Newell (2002); Hong and Newell (2008a,b); Hong (2010)]. The combined effects of task and environment systematically alter the unpredictability contained at the level of behavior. As demonstrated both in muscle force control (Hong and Newell, 2008a,b) and visual search and cognitive responses (Hong and Beck, 2010), difficult tasks and unpredictable environments resulted in predictable patterns of behavior. These studies demonstrated that patterns of variability changed systematically with the demands of the task and environment.

Our recent findings have begun to bridge the relationship between neural noise and behavioral variability (Hong et al., 2012a). We examined striatal local field potentials (LFPs) in the striatum of a transgenic mouse model of HD in comparison to wild-type controls. The LFPs were tracked while the mice were actively exploring a plus maze. By using an “event-related” approach to the analysis of the LFP patterns, we specifically targeted striatal activity 2 s prior to and 2 s after the mice entered the choice-point in the center of the maze. We found that the unpredictability or noisiness in the LFP signal declined as the mice approached the choice-point and this noisiness increased after the mouse made a decision and moved into an arm of the maze. HD mice exhibited a significantly higher level of unpredictability in their LFPs and a decreased number of 90° turns in the maze. Interestingly, we also found that the unpredictability of the LFPs was negatively correlated with the unpredictability of behavior. Thus, the noisier the signal, the more likely the mouse was to behave predictably, i.e., repeatedly performing the same action and running straight up-and-down a single arm. This phenomenon was consistent across both control and HD mice: both groups engaged in more predictable and repetitive patterns of behavior when the level of entropy in striatal LFP signals was higher.

We focused specifically on striatum due to its role in behavioral planning. But, if one considers activity in brain regions other than striatum to be reflected in the patterns of behavior, then our results suggest that the increased noise in the striatum in HD is what prevented the necessary unpredictability in the other regions that engendered behavioral flexibility in the controls. Thus, our study shows that behavioral inflexibility can be characterized through two different manifestations: (1) unwanted increases in variability and the inability to engage in a sustained behavior; and (2) large decreases in variability (e.g., perseveration) and the inability to transition (task-switch) to different behaviors. HD is a good example of the former, in which patients have difficulty staying still or maintaining a static posture. The dancelike movements or “chorea” arise out of the patient's control, akin to a system that is too easily perturbed into a different behavioral state. PD, on the other hand, leads to a “locked in” state, in which the neuromotor system is either too slow to transition to a new motor behavior (bradykinesia) or is unable to “escape” or transition out of a posture (dyskinesia/akinesia).

## Neurodegeneration and reduced ability to distribute neural information

The concept of entropy conservation developed for brain activation (Smotherman et al., 1996; Mandell and Selz, 1997) and behavior (Hong and Newell, 2008a,b; Hong and Beck, 2010) argued that adapting to different behavioral demands or environmental changes was the result of the redistribution of entropy or unpredictability. Our animal LFP data provided evidence of entropy conservation across brain and behavior. In a follow-up report (Hong et al., 2012b), we further expanded on the conceptual framework that unpredictability in neural signals is continuously being redistributed across the entire brain in order to allow the individual or animal to engage in different behaviors. We also presented entropy conservation as an explanation of the Garrett et al. (2011) findings where older individuals who performed more poorly on cognitive tasks exhibited a different distribution of variability across the brain than their young and high performing old subjects.

Our proposed theoretical framework of noise distribution remains consistent with the neuroanatomy literature that has long demonstrated a degree of functional specificity across brain regions. We also add to the existing view that neural noise is not necessarily a global phenomenon that describes the state of the entire brain. Rather, neural noise modulation is a dynamic process, where noise is consistently being passed from regions performing the primary function to remaining regions while a DA-GLU balance modulates noise levels. This process of noise redistribution would be governed by the conservation of entropy (Smotherman et al., 1996; Mandell and Selz, 1997), where the overall level of unpredictability in neural signals across the entire brain is held constant.

**Figure 2:**
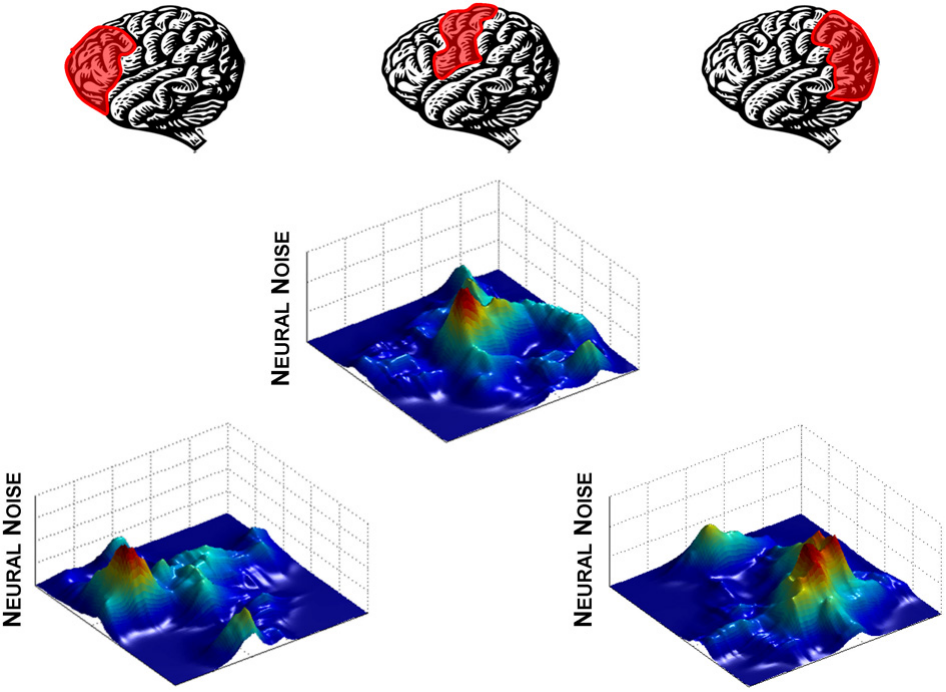
**Neural noise modulation across the brain viewed as landscape.** Here, levels of neural noise are represented as peaks and valleys on a landscape “map” of a brain unfolded as a two-dimensional surface. Noise is shifted from one brain region to another to alter the signal-to-noise ratio, depending on which region is playing a primary functional role.

Much like degeneracy in the modulation of neural noise through the DA-GLU relationship, distributing noise engenders flexibility in neural function. The entire neural system can remain relatively stable at a global level by maintaining a constant overall level of entropy across the brain (Smotherman et al., 1996; Mandell and Selz, 1997; Hong et al., 2012a,b), while having the flexibility to modulate signal-to-noise ratios in specific brain regions to perform different functions (Figure 2). This process does not require that signals from uninvolved regions to be “turned-off.” Instead, signals from these regions can be “shushed” by increased neural noise levels, effectively lowering the signal-to-noise ratio until the signal is no longer functional. Thus, switching a behavioral pattern or adapting to changes in the environment can be achieved by rapidly passing noise between different regions, allowing signals from regions performing primary functions to come to the fore.

## Inflexibility in neural noise distribution in aging and neurodegeneration

This redistribution process will become dysfunctional when noise levels can no longer be modulated effectively, which also restricts the number of available regions to “absorb” noise. As a result, aging and neurodegeneration reduces the ability to generate clearly distinguishable levels of variability in brain activation [as observed by Garrett et al. (2011)], consistent with the hypothesis of dedifferentiation in patterns of brain activation (Dennis and Cabeza, 2011). As a consequence of narrowing the range over which DA and GLU can be modulated, aging and neurodegeneration should also lead to inflexibility in the brain's ability to re-distribute neural noise. Empirically, this phenomenon will be observed as the dedifferentiation in the level of variability observed across all of the different brain regions.

**Figure 3:**
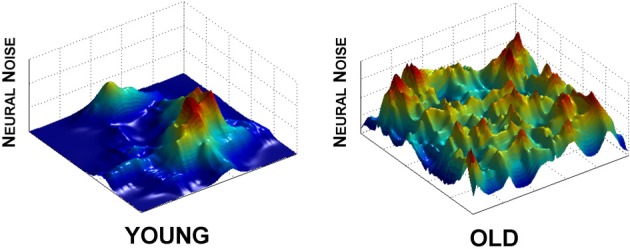
**Effect of aging on neural noise modulation.** As the solution manifold decreases in size, so does the ability to clearly delineate noise levels across the many regions of the brain. What results is a more even distribution of neural noise levels in the aging brain (old, right panel) in comparison to the clear peaks and valleys of the young (left panel). Effectively, this represents dedifferentiation across brain regions, but, reflected in neural noise levels. We used powernoise.m (Little et al., 2007) to generate the randomized patterns for these landscapes.

Within the entropy conservation framework, cognitive declines seen in poorly performing individuals would be a reflection of a decreased ability to shift variability across brain regions to perform a cognitive task. This viewpoint supports our HD data (Hong et al., 2012a) and also highlights the problem of variability redistribution in brain activation, in which the high level of neural noise in striatum reflects an inability to “pass on” the unpredictability signal to other regions (Figure 3). In the simplest sense, at any given moment in time, one brain region's signal becomes another brain region's noise. Such a noise redistribution process will allow the signal to be transmitted effectively across the entire brain while keeping the overall noise level constant.

Visually, this outcome would be reflected in a more even distribution of noise across the brain, where specific functional areas become nearly indistinguishable from those that are not (Figure 3, right panel). The interesting and important difference in our hypothesis is that dedifferentiation in neural activation is not exclusively a problem of signal transmission, but that dedifferentiation of noise levels also plays a key role in the development of behavioral inflexibility and other clinical symptoms of aging and neurodegeneration. In sum, as the ability to shift noise levels across the brain becomes deficient, the brain's ability to transition from one behavior to another also reduces its ability to modulate behavior appropriately.

## Summary and conclusions

The literature reviewed in this paper converges on the hypothesis that inflexibility is a key problem in aging and neurodegeneration. Instead of behavioral inconsistency, inflexibility is characterized by: (1) reduced ability to transition from one behavior to another; and (2) inability to adapt to perturbations or challenges in the environment. Healthy nervous systems are able to perseverate or focus their behaviors, generating repetitive patterns of variability, or producing less correlated or scattered behavioral sequences as necessary, depending on task demands, and environmental conditions. Unhealthy neural systems, on the other hand, generate more inconsistent behaviors when focus is required and perseverate when variability is needed for exploration or adapting to the environment.

The problem of inflexibility, however, is not restricted to behavior alone. We have also presented the argument that for both aging and neurodegeneration, inflexibility is a problem at multiple levels of the nervous system. Inflexibility in neural communication is evident in the dedifferentiation of levels of variability in BOLD signals across the brain, as observed by Garrett et al. (2011). We propose that this result is a reflection of an inability to redistribute noise levels, a process that would normally allow the brain to selectively “attend” to signals from regions primarily involved in the task, while “extinguishing” the signal in non-involved areas. With aging and neurodegeneration, this process becomes restricted as clear highs and lows in neural noise gradually disappear, leaving a fairly constant level of noise across the entire brain. Thus, increased noise alone is not the sole marker of disorder; the more appropriate marker, instead, would be evenly distributed levels of noise across the brain.

We have proposed that in the aging or degenerating brain the ability to effectively redistribute neural noise levels by modulating DA and GLU levels becomes restricted as: (1) DA neurons are lost; (2) GLU uptake is restricted; and (3) the DA-GLU relationship is altered.

These neurobiological changes reduce the range of solutions over which neural noise can be modulated, which restricts the brain to a narrower range of noise levels. Such narrowing of neurotransmission would prevent the highs and lows in noise level and brain activation variability characteristic of a healthy nervous system.

This does not mean, however, that DA and GLU are exclusively responsible for modulating neural noise or that no other neurotransmitters are involved. Rather, it is quite likely that other neurotransmitters such as norepinephrine and GABA are likely to play a role within a complex network that modulates neural noise levels and are altered as a function of aging and neurodegeneration. This network remains to be uncovered. Because the goal of this paper is to present a theoretical framework for understanding the problems of variability in aging and neurodegeneration, we have delimited our discussion to DA and GLU. This is because DA and GLU have been widely studied in aging and neurodegeneration, forming an evidence-base that is sufficiently large, insofar as to support the development of the current theoretical framework. Further research using novel paradigms is needed to expand the understanding of the role of not just one or two neurotransmitters in aging and neurodegeneration, but instead, employs paradigms in order to gain insight into the interaction between groups of neurotransmitters.

Clinically, this conceptual framework raises questions as to how the effects of aging and neurodegeneration can be attenuated or prevented. Answers are currently difficult to come by as because both excessively high and low synaptic levels lead to neural dysfunction and existing pharmacological therapies are designed to either be agonists or antagonists to DA and GLU. Moreover, maintaining the delicate dynamic balance between DA and GLU (and possibly other neurotransmitters) is a key component of restoring flexibility by controlling the distribution of neural noise levels across the brain, something that cannot yet be achieved through pharmacological treatments currently in existence. Improving behavioral symptoms requires the development of therapies that: (1) dynamically modulate synaptic DA and GLU; (2) restore the balance between them; (3) reverse the declines in DA and GLU function; or (4) directly improve the redistribution of electrical activity and noise across the brain (e.g., transcranial magnetic stimulation or deep brain stimulation). It also remains an open question as to whether multi-level, brain-behavior therapeutic approaches could yield better results than targeting the brain directly. It is clear that many unknowns still exist, emphasizing the need for more research aimed at developing new tools to address the problems associated with aging and neurodegeneration.

### Conflict of interest statement

The authors declare that the research was conducted in the absence of any commercial or financial relationships that could be construed as a potential conflict of interest.
